# Glioblastoma: Relationship between Metabolism and Immunosuppressive Microenvironment

**DOI:** 10.3390/cells10123529

**Published:** 2021-12-14

**Authors:** Ainhoa Hernández, Marta Domènech, Ana M. Muñoz-Mármol, Cristina Carrato, Carmen Balana

**Affiliations:** 1 B·ARGO (Badalona Applied Research Group of Oncology) Medical Oncology Department, Catalan Institute of Oncology Badalona, 08916 Badalona, Spain; ahernandezg@iconcologia.net (A.H.); mdomenechv@iconcologia.net (M.D.); 2 Pathology Department, Hospital Universitari Germans Trias i Pujol, 08916 Badalona, Spain; ammunoz.germanstrias@gencat.cat (A.M.M.-M.); ccarrato.germanstrias@gencat.cat (C.C.)

**Keywords:** glioblastoma, metabolism, microenvironment

## Abstract

Glioblastoma (GBM) is the most aggressive brain tumor in adults and is characterized by an immunosuppressive microenvironment. Different factors shaping this tumor microenvironment (TME) regulate tumor initiation, progression, and treatment response. Genetic alterations and metabolism pathways are two main elements that influence tumor immune cells and TME. In this manuscript, we review how both factors can contribute to an immunosuppressive state and overview the strategies being tested.

## 1. Introduction

Glioblastoma represents the most prevalent and malignant primary tumors of the central nervous system (CNS) in adults. Currently, the available treatment options are limited and based on standard surgery and radio-chemotherapy with alkylating agents and the addition of tumor-treating fields (TTFields), an accepted treatment in several countries [1,2,3]. The prognosis for this type of tumor remains poor with an overall survival time of 14–21 months depending on the studies [4]. Although numerous diagnostic or predictive of response biomarkers have been identified, such as isocitrate dehydrogenase (*IDH*) mutation or O6-methylguanine DNA methylation status [5], treatment has not improved in parallel over the last three decades [5]. Therefore, there is an urgent need to develop novel and effective treatment strategies.

Over the last several years, the role of the relationship between tumor metabolism and immune regulation of the microenvironment has become recognized as an important factor involved in tumor growth and progression. The brain is a highly metabolically active organ, dependent on glucose as its main energy substrate. However, other substances, such as fatty acids or amino acids, can be used as a source of energy [6,7,8]. Furthermore, the cells that form the brain, such as astrocytes, neurons, and microglia influence each other’s nutrient uptake, demonstrating the importance of different cell types in brain metabolic homeostasis. A better understanding of the brain tumor microenvironment, as well as the metabolism of brain tumors, will undoubtedly guide effective therapeutic strategies. In this review, we describe how the relationship between TME, tumor metabolism, and genetic alterations are fundamental to the initiation and progression of GBM and provide an overview of strategies currently being tested.

## 2. Tumor Microenvironment in Glioblastoma

The brain has long been recognized as an “immune privileged” organ because of the restrictions imposed by the blood-brain barrier (BBB), in addition to the lack of a lymphatic system [9]. The immune privileged concept was discarded in 2015 after the discovery of functional lymphatic vessels in the meninges with a direct drainage pathway to the cervical lymph nodes [10]. Now, the brain is proposed to be an immunologically distinct rather than a privileged organ [11].

Brain tumors, as well other tumors, modify the phenotype of stromal cells, creating a tumor microenvironment that favors tumor development and progression. The BBB is damaged by inflammatory stimuli and is often characterized by abnormal vasculature, allowing the flow of circulating myeloid and lymphoid cells, normally absent in the normal brain parenchyma [12]. GBM is a heterogeneous tumor, and its tumor microenvironment is composed not only of tumor cells, but also of non-neoplastic cells such as immune, vascular, and other glial cells [13]. Gliomas are considered a cold tumor due to the low numbers of tumor-infiltrating lymphocytes (TILs) and other immune effector cell types [14].

In recent years, single-cell RNA-sequencing (scRNAseq) studies have acquired a higher relevance in the study of TME. Bulk tumor analysis allows the study of the genetic status of tumor cells, the expression profiles of the various cells within each tumor, limiting the view of the interaction of tumor cells with the TME. scRNAseq can help solve these problems. However, it has its drawbacks given the high cost and logistics required for its development.

scRNAseq enables the study of the biological properties of individual cells with an unprecedented resolution. In the first scRNAseq studies, between 10 and 100 cells were analyzed and characterized [15,16,17]. Currently, transcriptomes of up to tens of thousands of individual cells can be analyzed and sequenced in a single project [18]. For this reason, it is the reference technology for the quantification and phenotyping of the TME as well as characterizing its heterogeneity [19,20,21].

Understanding the interaction between brain tumor cells and the other cells of its microenvironment is of fundamental importance and plays a key role in tumor growth and identification of therapeutic targets’ response to treatment. Caruso et al., [22] exploit single-cell data developing single-cell Tumor–Host Interaction (scTHI), a tool to identify the Ligand-Receptor pairs that modulate the tumor microenvironment cross-talk in glioma. scTHI is based on the hypothesis that when patterns of interaction are active, they are also simultaneously and highly expressed in homogeneous cell populations. Their results suggested shared cross-talk mechanisms that exist in glioma due to unexpected interaction partners being are highly conserved in most tumor samples.

In this section, we will differentiate between the non-immune cellular components and immune cellular components focusing on the latter.

### 2.1. Non-Immune Cellular Components

#### 2.1.1. Vasculature

The brain’s vasculature is composed of a complex network of blood vessels that provide blood flow and maintain the integrity of the BBB. However, the BBB loses its integrity in malignant brain tumors such as GBM, being one of the most vascularized tumors with extensive neo-angiogenesis. However, this vasculature is abnormal and disorganized forming hyperdilated and permeable vessels, a common hallmark of this type of tumor [23]. The vascular abnormalities are predominantly due to highly elevated levels of vascular endothelial growth factor (VEGF) [24].

#### 2.1.2. Glioma Stem Cells (GSC)

Tumors are composed of small populations of cells called cancer stem cells with stem-like properties, such as self-renewing capacity or differentiation [25]. This cell type was identified in brain tumors by Singh in 2003 [26] and, later, other studies using single surface marker approaches also identified them [27,28]. It has been suggested that GSC can differentiate into endothelial cells within the glioma vasculature [29,30], facilitating GSC reservoirs in the perivascular niche (PVN) to remain isolated and proliferate safely [31]. Furthermore, GSC initiates, supports, and maintains tumor growth and promotes angiogenesis.

#### 2.1.3. Glial Cells and Neurons

Glial cells provide structural support in the brain by maintaining homeostasis. They are the most abundant cell in the CNS, accounting for approximately 80–90% of the total and two types are distinguished: astrocytes and oligondendrocytes. While oligondendrocytes are responsible for myelin formation, the astrocytes are usually localized to the PVN and play an important role in maintaining the BBB [32]. In addition, astrocytes have pro-tumoral functions, secreting neurotrophic factors that promote glioma cell proliferation [31].

Neurons are a brain-specific cell type, like astrocytes, which are thought to contribute to the creation and growth of tumors.

### 2.2. Immune Cellular Components

#### 2.2.1. Microglia/Macrophages

Microglia are the resident macrophages of the CNS, found in all regions of the brain, and constitute the first line of innate immune defense in the CNS [33]. Microglia develop from embryonic yolk sac progenitor cells and migrate to the CNS early in development to mature into different populations of CNS monocytes and they are not replenished postnatally [34]. They are ontogenetically distinct, but indistinguishable from peripheral bone marrow-derived macrophage infiltrate after activation by glioma cells. Several studies have attempted to distinguish microglia from invading monocytes, using bone-marrow chimeras and cell surface antibodies, with discrepancies in the results [35].

Together, microglia and macrophages are known as tumor-associated macrophages (TAMs), accounting for up to 30% of the tumor mass being the main immune cells [36]. They are critical for gliomagenesis and continued tumor growth. Microglia depletion reduces glioma growth in experimental GBM models [35]. Several factors released by microglia promote glioma proliferation and/or migration. Stress-inducible protein 1 (STI1), a cellular prion protein-ligand, increases the proliferation and migration of GBM in vitro and in vivo [37]. Others, such as epidermal growth factor (EGF), stimulate glioblastoma cell invasion [38], or transforming growth factor-β (TGF-β) that increases the migration of glioma cells [39].

As well as macrophages in other tissues, TAMs change their phenotype depending on the type of stimulus they receive from the environment [40]. Traditionally, two TAM phenotypes have been described: M1 macrophages or pro-inflammatory/antitumoral and M2 Macrophages or anti-inflammatory/pro-tumoral [41]. The M1 phenotype is acquired after stimulation with Toll-like receptor 4 (TLR4) ligands and interferon gamma (IFN-γ), but the M2 phenotype occurs after stimulation by interleukins 4, 10, and 13. The anti-inflammatory M2 phenotype has been associated with the promotion of tumor growth. Moreover, the M2 subtype can be subdivided into M2a (Th2 responses, type II inflammation, killing of pathogens, allergy), M2b (Th2 activation, immunoregulation), and M2c (immunoregulation, matrix deposition, tissue remodeling) activation states [35]. It appears that the full spectrum of TAM is much more diverse and dynamic. scRNAseq demonstrated a gradual change of three transitional states in TAMs [19]. The pattern first started with a microglia phenotype (P2RY12+/TMEM119+), then it turned into a polarized macrophage (CD163+), and finally converged into M2b macrophages (IL1RN+) with the activated expression of strong angiogenesis signaling molecules (VEGF-A).

Furthermore, there is strong evidence between poor survival and the increased macrophage density in different types of cancer, such as thyroid or lung [42,43]. Szulzewsky et al., using RNA microarrays analyses, observed that the differential expression of approximately 1000 transcripts was twofold higher in microglia and glioma-associated macrophages relative to control microglial cells [44]. In addition, Zeiner et al., conducted analyses to determine the relationship between the survival of GBM patients and the expression of specific M1 or M2 polarization markers. CD74, an M1 polarization marker, correlated positively with increased patient survival [45].

#### 2.2.2. Tumor-Infiltrating Lymphocytes (TILs)

Lymphocytes are cells that play an important role in the adaptive immune system. They are produced in the bone marrow and mature in the thymus from which mature T cells are released to peripheral lymphoid organs where they are primed by engaging with professional APCs. In a pathological state, such as GBM, T cells leave the circulation and enter inflamed tissues, this mechanism is well characterized [46].

T cells are the primary lymphoid component of the GBM TME, but they constitute less than 0.25% of cells in total [47]. They may exert both pro- and anti-tumor functions in TME and several types can be distinguished, such as CD4^+^ T helper (Th), CD8^+^ T cytotoxic (Tc), and Treg. Tregs are potent suppressors of the adaptive immune response through their ability to inhibit the proliferation of any cytokine-secreting effector T cells.

The peculiar immune environment of the brain can limit the activity of T cells in GBM by a low number of antigen-specific TILs and exhausted phenotypes [48].

Fu et al. [49] analyzed, via single-cell study with mass cytometry, infiltrating immune cells from initial and recurrent GBM surgical tissues, both of which were coupled with their paired peripheral blood mononuclear cells. They observed the following findings: (1) The T cell population exhibited a complex diversity based on their surface with high expression of PD-1, LAG, 3, TIM-3, and IDO in some T cell subgroups; (2) Treg proportions in the tumor lesions were significantly increased across all patients; (3) PD-1+, TIM-3+, or LAG-3+ T cells are recognized as exhausted subsets, and (4) the proportions of exhausted CD4^+^ and CD8^+^ T cells were distinctly higher at the tumor sites.

Furthermore, the CD4^+^ and CD8 ^+^ populations increase with tumor grade [50] and may correlate with poor survival outcomes [47].

#### 2.2.3. Natural Killer Cells

Natural Killer (NK) cells are a type of lymphocyte produced in the bone marrow, whose effector function is mediated by cytokine production and cytotoxic activity. Their function is often affected by immunosuppressive factors released by tumor cells such as highly expressed major histocompatibility complex Class I molecules which act as ligands for inhibitory receptors expressed on NK cells [51].

#### 2.2.4. Neutrophils

Neutrophils are the most abundant type of granulocytes in humans and constitute around 70% of total leukocytes in the body. They form an essential part of the innate immune system [52]. Commonly, they are located in the GBM tumor core [53].

In recent years, interest in neutrophils as a critical component of TME has grown because of its prognostic value. Most glioma patients have strong neutrophilia, as do other cancer patients [54]. Several studies demonstrated that the number of circulating and infiltrating neutrophils correlates with poor prognosis. Mason et al., assessed altered neutrophil-lymphocyte ratio (NLR) in a retrospective review of patients with newly diagnosed GBM treated with radiotherapy and concomitant temozolomide. They observed that elevated NLR may predict worse outcomes [55]. Furthermore, NLR is correlated with glial tumor grade. Zadora et al., evaluated preoperative NLR in a cohort of neurosurgical patients treated for glial brain tumors. The preoperative NLR was analyzed in accordance with WHO glial tumor classification, which distinguishes G1, G2, G3, and G4 (glioblastoma) tumors. The highest value of NLR was observed in G4 and was significantly higher compared with G3, G2, and G1 [56]. Other studies, including studies with RNA sequencing data, have also shown the same results [57,58,59,60].

Furthermore, a high peripheral neutrophil count prior to treatment correlates with a positive initial response to the vascular endothelial growth factor A (VEGF-A) antibody, bevacizumab [61]. However, increased neutrophil infiltration of tumor tissue is associated with a higher grade of glioma in later stages and acquired resistance to treatments [62]. Neutrophils directly promoted GBM-initiating cell proliferation and migration via the production of S100A4, which induced transition to a mesenchymal phenotype, favoring cancer invasion and resistance to anti-VEGF therapies [62].

## 3. Genomic and Epigenomic Alterations in Glioblastoma

To better understand the determinants of GBM progression, several systems have been proposed for classifying glioblastoma into molecular subtypes [63,64,65,66,67,68,69,70,71,72]. The molecular classification of GBM is a recent tool that can complement the traditional pathology-based description. In fact, in 2016, by combining morphology and genetic alterations, the WHO updated their guidelines, leading to the emergence of two entities based on the mutational status of the *IDH* gene: *IDH* wild-type and *IDH* mutated GBM [73].

Multi-omics studies from the landscape of GBM in the Cancer Genome Atlas Research Network (TCGA) and the Chinese Glioma Genome Atlas (CGGA) revealed the complicated genetic profile of GBM [74,75]. The somatic aberrations, such as *IDH* and *TP53* mutations, *EGFR* (epidermal growth factor receptor) gene amplification, *TERT* (Telomerase reverse transcriptase) promoter mutations, *PTEN* (Phosphatase and tensin homolog) mutations, and *ATRX* (Alpha thalassemia/mental retardation syndrome X-linked) mutations enabled improved diagnosis and can help determine the prognosis and identify the optimal therapy for specific subgroups.

The complex genetic profile of GBM is also due to the wide range of chromosomal changes [67] or significant mutations [74]. The most frequent mutations include *TP53, EGFR*, *PTEN*, *NF1* (neurofibromatosis 1), *PIK3CA* (Phosphatidylinositol 4,5-bisphosphate 3-kinase catalytic subunit alpha isoform), *RB1* (Retinoblastoma-associated protein 1), *CDKN2A* (Cyclin-dependent kinase inhibitor 2A) deletion, or *PDGFRA* (Platelet-derived growth factor receptor) [66,67,74].

It is important to bear in mind DNA (deoxyribonucleic acid) methylation states in GBM because of the correlation with survival [76]. The status of methyl-guanine-methyl-transferase (*MGMT*) is a prognostic factor for GBM patients and has a significant correlation with worse survival rates [67]. Noushmehr et al., first described the cytosine-phosphate-guanine (CpG) island methylator phenotype (G-CIMP) and, later, Brennan et al., identified *MGMT* methylation as a predictive factor for response to temozolomide [65,67].

Expression profiling of GBM tumors identified four subtypes: proneural (PN), neural (NEU), classical (CL), and mesenchymal (MES) [66]. Later, Wang et al. [77], suggested that the NEU phenotype is non-tumor specific but rather a contamination, thus explaining why the neural subtype was the only subtype to lack characteristic gene abnormalities.

The PN subtype is characterized by *IDH1* mutations and/or *PDGFRA* amplification, is found mainly in younger patients, and may have better survival rates. The NEU subtype, characterized by the expression of neuron markers similar to normal brain tissue has a good response to radiation and chemotherapy. The CL subtype with *EGFR* amplification and/or mutations has the best response to chemoradiotherapy, with a dramatic reduction in mortality. MES with *NF1* loss and/or mutations has the worst prognosis among all the subtypes [66,78].

### Correlation with Tumor Microenvironment

Given the differences between GBM subtypes, it is very important to know whether there are differences in the respective TMEs.

The analysis of TCGA gene expression data for GBM showed that mRNA expression for different cytokines, immune cell markers, and immune-associated signaling pathways was increased in the mesenchymal subtype, suggesting that this was the most pro-inflammatory [79]. Later, Wang et al., by performing in silico analysis, found that the MES subtype was enriched in M2 macrophages and neutrophils [77]. Other studies have observed the same results. Carrato et al., observed that immunophenotyping of the MES subtype exhibited higher positive effector and suppressor cell score, and lower levels of immune checkpoint molecules. Moreover, the cell-type deconvolution analysis revealed that these tumors are highly enriched in M2 macrophages, resting memory CD4^+^ T cells, and activated dendritic cells [80].

In addition, Wang et al., described that the *NF1* gene is associated with the recruitment of TAMs [77]. By contrast, the presence of *IDH1/2* mutations is associated with a limited number of immune cells [81] as well as a PN subtype [77,82]. *EGFR* alterations have been linked to vasculature modifications facilitating the recruitment of immune cells in brain tumors [83,84].

## 4. Metabolism in Glioblastoma

The brain is a highly metabolic organ and consumes 25% of the body’s glucose. Glucose is the main energy substrate, however, other metabolites such as amino acids, fatty acids, or lactate are also used as a source of energy [6,85].

Gliomas arise in a hypoxic environment, being forced to modify their metabolic pathways to obtain nutrients [86]. Altered cellular metabolism is a relevant hallmark of gliomas and genetic alterations are the cause of these deviations.

### 4.1. Metabolic Pathways

#### 4.1.1. Aerobic Glycolysis

Glycolysis is the metabolic pathway by which glucose is broken down into two molecules of pyruvate, while producing energy in the form of ATP and NADH. One of the best-known alterations in tumor cell metabolism is the capacity for aerobic glycolysis first described by Otto Warburg in the 1920s [87]. The Warburg effect is defined by two points in the context of oxygen availability: (i) increased glucose consumption by the tumor, and (ii) conversion of glucose to lactate outside the mitochondrion as opposed to mitochondrial oxidative phosphorylation in normal cells. It also promotes biosynthesis by providing the macromolecules necessary for the synthesis of DNA and lipids essential for tumor growth, termed anabolic metabolism [88], which is so relevant in GBM [89].

#### 4.1.2. Amino Acids Metabolis

Different amino acids are metabolized by GBM cells, with tryptophan being the best-characterized pathway.

##### Tryptophan

Tryptophan is an essential amino acid, a precursor of neurotransmitters such as serotonin and melatonin. In addition, tryptophan can be metabolized to kynurenine by two enzymes, indoleamine 2,3-dioxygenase 1/2 (IDO1/2) and tryptophan 2,3 dioxygenase (TDO), generating nicotinamide adenine dinucleotide (NAD+) [90]. Kynurenine is subsequently metabolized via several pathways to produce other metabolites such as kynurenic acids, quinolinic acids, anthranilic acid, etc. [91].

##### Glutamine and D2-Hydroxyglutarate Metabolism (2HG)

Glutamine is the most abundant amino acid in the human body, it is an excitatory neurotransmitter and is involved in numerous intermediary metabolic processes, especially in the synthesis of amino acids and purines, the tricarboxylic acid cycle (TCA cycle), and the generation of urea. In the TCA cycle, also known as the Krebs cycle, energy is released via the oxidation of acetyl-CoA derived from macromolecules (carbohydrates, lipids, and proteins) into carbon dioxide and chemical energy in the form of ATP. In addition, the cycle provides precursors of certain amino acids, as well as the reducing agents NADH and NADPH [92].

Intratumoral glutamine levels in GBM are increased when compared with normal brain tissue [93,94]. Glutamine comes from two main sources: astrocyte-derived glutamine using the glutamine transporter ASCT2 present in glioma cells and a small fraction comes from the systemic circulation. In addition, when glioma cells are depleted of glutamine, they regulate the conversion of glutamate into glutamine by upregulation of the enzyme glutamine synthetase (GS) [95].

**Figure 1:**
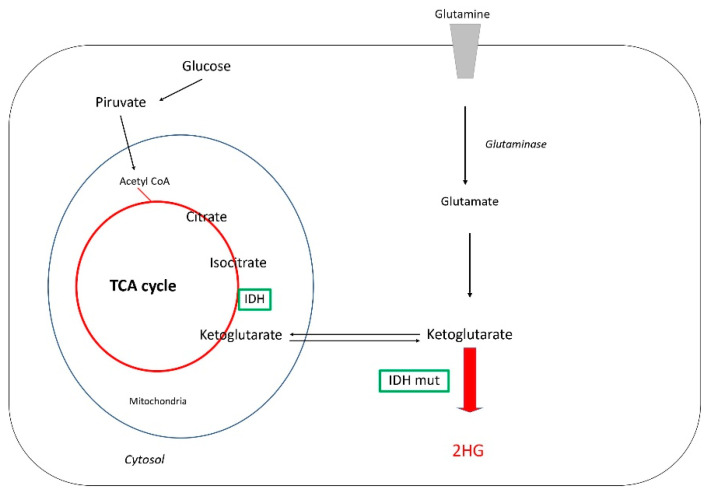
Glutamine and D2-Hydroxyglutarate metabolism in *IDH* mutant.

It is noteworthy, that the role of glutamine metabolism in glioma has become more important with the discovery that glutamine can give rise to the oncometabolite 2HG in gliomas that have *IDH1/2* mutants [96]. 2HG is the unique immune metabolomic pathway found in many cancer cells. Both *IDH1* and *IDH2* catalyze the decarboxylation of citrate using NADP+ and produce alfa ketoglutarate (alpha-KG). Alpha-ketoglutarate acts as a substrate for an alternative reduction reaction that incompletely reduces it to 2HG instead of isocitrate like NADPH (Figure 1).

##### Other Amino Acids: Adenosine and Arginine

Adenosine plays an important role in biochemical processes, such as energy transfer, in the form of ATP and ADP, as well as being a signal transducer in the form of cyclic adenosine monophosphate (cAMP). Furthermore, it plays an important role as a neuromodulator in the central nervous system. In normal physiology, adenosine and ATP are found in the cytosol, while at the extracellular level they are rarely observable [97]. In gliomagenesis, intracellular adenosine can be secreted bidirectionally and ATP liberated extracellularly induced by inflammation or hypoxia [98].

Arginine is a semi-essential amino acid with different functions. Its serves as a precursor for the synthesis of nitric oxide, proteins, polyamines, and urea [99]. Arginine is a substrate for arginase 1 (ARG1), which converts it to urea and ornithine, and cytokine-inducible nitric oxide synthase (iNOS), which converts it to citrulline and nitric oxide (NO). In GBM, a high accumulation of arginine by-products from arginine metabolism is evident due to the abundance of arginine transporters [89]. This suggests that arginine metabolism is functional and that it may be sensitive to selective depletion.

#### 4.1.3. Lipid Metabolism

Lipids are essential to the structure and function of the brain. Indeed, it is the organ with the highest cholesterol content in the body. Cholesterol is mainly synthesized locally by astrocytes because it does not cross the BBB [100].

Other lipids found in large quantities in the brain are sphingolipids. Their biological importance lies in the cell signaling role they effect. Two of the most studied are ceramide, which is involved in the regulation of apoptosis, and sphingosine-1-phosphate (S1P), which plays a role in survival, migration, and inflammation [101]. Ceramide is obtained de novo via ceramide synthase (CERS1-6) but can also be produced via salvage following the breakdown of complex sphingolipids [102]. Sphingosine kinase 1 and 2 (SK) are enzymes that regulate the levels of ceramide and S1P.

In recent years, the interest in S1P has grown in GBM due to its important role as a signaling molecule, able to stimulate proliferation, motility, migration, and survival [102,103]. S1P is formed intracellularly from sphingosine by two isoenzymes SK 1 and 2. The S1P phosphatases 1 and 2 (SGPP1) dephosphorylate S1P back to sphingosine whereas S1P lyase (SGPL) mediates the irreversible cleavage to hexadecenal and phosphoethanolamine [104].

S1P levels in glioma tissues were higher than in normal brain tissue [105]. Likewise, the levels found in surgical specimens of glial tumor (low and high-grade malignancy) revealed an inverse correlation between the amount of ceramide and tumor malignancy [106]. Abuhusain et al. [105] observed sphingolipid metabolism favoring S1P over ceramide in GBM tissues compared with normal gray matter, and increased S1P content in the tumors significantly correlated with increased SK) and decreased SGPP2 expression. Moreover, the inhibition of S1P production by cultured GBM cells, using a highly potent and selective SK1 inhibitor, blocked angiogenesis in co-cultured endothelial cells without affecting VEGF secretion.

This altered ceramide/S1P balance seems to be important and may be an opportunity for the development of new therapies such as antiangiogenic agents.

### 4.2. Correlation with Genomic Alterations

Genomic alterations are associated with metabolic adaptation allowing the proliferation and survival of tumor cells but, currently, this is incompletely understood. Both, *IDH* mutations and growth factor receptor tyrosine kinase (RTK) encoding genes have become important in recent years.

#### 4.2.1. Receptor Tyrosine Kinase Amplification

Gain-of-function changes in the growth factor signaling system are among the most frequent genetic alterations in glioma. RTKs, especially epidermal growth factor, are commonly amplified in GBM by over 50%, including approximately half of these carrying the gain of function *EGFR* variant III (EGFRvIII) alteration [107].

The PI3k-Akt-mTOR pathway has a critical regulatory role in energy metabolism in neurons and glia [108] signaling downstream of amplified RTKs. RTK signaling through PI3K–AKT and both mTORC1 and mTORC2 increases *MYC* expression. The amplified oncogenes can coexist with mutations in genes encoding *PI3k* and with loss of *PTEN* [67]. This situation may result in the reprogramming of the cellular metabolism by engaging AKT–mTOR signaling [109]. Data suggest a central role for mutated *EGFR* in glycolysis and lipogenesis in the pathogenesis of GBM. Mutant *EGFR* drives GBM glycolysis by *AKT*-dependent and independent pathways via *MYC* and mTORC2 and drives fatty acid and cholesterol synthesis [109,110,111]. *EGFR* may also be involved in amino acid metabolism by phosphorylation of the cystine-glutamate antiporter xCT regulated by mTORC2 [112]. The biological function of *MYC* in cell proliferation and metabolism has been well established [113], but it is rarely amplified in adult GBM [67]. *MYC* controls the expression of glucose metabolism genes such as glucose transporter (*GLUT 1*), located in the blood-brain barrier, simulating the Warburg effect [114]. Additionally, *MYC* increases intratumoral glutamine levels in GBM [92,95].

#### 4.2.2. Isocitrate Dehydrogenase (*IDH*) Mutations

*IDH* is a carbohydrate metabolism enzyme involved in the Krebs cycle that catalyzes the oxidative decarboxylation of isocitrate to form 2-oxoglutarate [115].

*IDH* mutations are early events in gliomagenesis [116,117]. They have been observed in 5% of GBM and 70–80% of low-grade gliomas, with the *IDH1* mutation being the most frequent, accounting for more than 95% of cases [118,119]. Both *IDH1* and *IDH2* mutations play an important role in several cellular functions, such as glucose sensing, glutamine metabolism, lipogenesis, and regulation of the cellular redox state.

In 2009, Dang et al., discovered that *IDH1* mutations carried a gain of function that reduces alpha-KG to produce oncometabolite (2HG) [96]. *IDH* is an NADP+ dependent enzyme that interconverts isocitrate and alpha-KG [120]. Consequently, 2HG production impairs the normal biosynthetic pathways of *IDH* activity to convert isocitrate to alpha-KG and generate NADPH by altering the metabolic flux of alpha-KG and depleting NADPH. Glutaminolysis represents a compensatory mechanism in *IDH*-mutated gliomas to maintain the necessary level of metabolites while producing abundant amounts of 2HG [121]. The accumulation of 2HG increases the oxidative stress present in cancer cells and increases reactive oxygen species (ROS), promoting tumor growth [122]). Moreover, the increased synthesis of alpha-KG via the mitochondria produces a reduction of alpha-KG substrates, such as citrate. Citrate is fundamental for acetyl-CoA synthesis and fatty acid formation.

#### 4.2.3. Other Genomic Alterations

The *TP53* gene is the most frequently mutated gene in human cancer [123]. A *TP53* mutation occurs in about 30% of primary GBM cases, however, it is found in 65–90% of cases of secondary GBM [124]. This tumor suppressor gene has key roles in responding to DNA damage, hypoxia, and oncogenic activation. A novel function of p53 has emerged, showing its potential to regulate cellular metabolism and oxidative stress. One of the ways in which p53 functions is by slowing down glycolysis and promoting oxidative phosphorylation, providing a mechanism for blocking the Warburg effect [125]. Research in this area is currently underway.

GBM with loss of *PTEN* activity has an elevated expression of the glycolytic enzyme hexokinase 2 (HK2). HK2 is an important facilitator of aerobic glycolysis in GBM, enabling survival and proliferation of the tumor microenvironment. High expression of HK2 predicts poorer overall survival [126].

## 5. The Role of Metabolism in the GBM Microenvironment

GBM is characterized by heterogeneous and immunosuppressive TME. The interaction between brain tumor cells and the other cells of its TME is regulated, at least in part, by tumor metabolism. Alterations in tumor cell metabolism, remodeling biological cell processes, contribute to progression. We explain how altered metabolic pathways in GBM contribute to tumor growth and immunosuppression.

### 5.1. Aerobic Glycolysis

The immune cells, in particular effector T cells, are dependent on glycolysis [127]. Glycolysis supports the proliferation and effector functions of T cells, and their exhausted phenotype is related to low glucose availability [128]. Exhausted T cells are refractory to checkpoint inhibitor therapy and maintain an immunosuppressive microenvironment. Another factor contributing to immunosuppression is lactate accumulation. Lactate has been shown to polarize macrophages towards the M2/pro-tumoral phenotype [129].

The glycolytic pathway also controls the functionality of neutrophils, decreasing their effectiveness [130]. NK cells undergo metabolic reprogramming due to the direct limitation of the rate of glycolysis is sufficient to inhibit IFN-γ production and granzyme B expression [131]. Both mechanisms contribute to an inefficient immune response.

### 5.2. Amino Acid Metabolism

Several studies demonstrated that tryptophan metabolism induces a state of immunosuppression [132,133]. The upregulated expression of IDO1 produces low tryptophan levels, resulting in anergy in effector T cells [133]. This pathway can induce Treg differentiation based on the activation of the aryl hydrocarbon receptor (AHR), a cytoplasmic transcription receptor, via kynurenine [134]. Furthermore, AHR activation reduces proliferation and infiltration of effector T cells, decreases inflammatory cytokines, such as IFN-gamma or IL-17, and regulates IL-1 Beta, facilitating the conversion of naïve CD4 to the Treg suppressor immunophenotype [135,136].

Arginine is a modulator of immune function promoting tumor progression and immunosuppression. Macrophages are the immune cell subset most influenced by arginine metabolism and present different phenotypes, M1 or M2, depending on how the arginine is metabolized [137]. TAMs which metabolize arginine via iNOS express the M1 phenotype/anti-tumor and, conversely, TAMs which use ARG1 have a more anti-inflammatory/M2 phenotype.

Adenosine has been identified as a potent anti-inflammatory. Different roles in neutrophils, lymphocytes, and macrophages have been described. One mechanism is its interaction between regulatory and effector T cells. Adenosine binds the adenosine receptor, which is upregulated in activated effector T cells, causing immunosuppression [138]. The main role is to stimulate the proliferation and migration of endothelial cells and vascular endothelial growth factor-mediated angiogenesis [139].

The overaccumulation of 2HG can inhibit anti-tumor immunity and promote tumor growth. 2HG inhibits T cell activity by inhibiting enzymes such as ornithine decarboxylase. Furthermore, it directly stops T cell activation by inhibiting calcium influx [140]. The suppression effect is most considerable in CD4^+^ T cells.

### 5.3. Lipids

Altered phospholipid metabolism induces tumor proliferation, sequestering T cell populations away from the tumor microenvironment. GBM causes sequestration of T cells in bone marrow via T-cell internalization of the sphingosine-1-phosphate receptor 1 (S1PR1). S1P acts as a chemotaxis inductor for innate and adaptative immune cells [141] and increased GBM-derived S1P promotes the formation of TAMs. In turn, TAMs increase SK1 activity [103].

## 6. Therapeutic Opportunities

In recent years, advances in immunotherapy have revolutionized the treatment of cancers such as non-small cell lung cancer and melanoma, but not glioblastoma [142]. One of the difficulties encountered in glioblastoma immunotherapy is its own TME. The different metabolic pathways involved in maintaining immunosuppression and glioblastoma outgrowth in TME and immunometabolism represent a unique opportunity to develop therapeutic strategies.

### 6.1. IDO Inhibitors

One of the most advanced strategies involving tryptophan metabolism is the IDO inhibitors. Currently, several IDO inhibitors are undergoing clinical evaluation.

PF-06840003 is a highly selective IDO1 inhibitor with single-dose daily administration. In preclinical studies with mice carrying tumor grafts, PF-06840003 reduced intratumoral kynurenic levels and inhibited tumor growth in both monotherapy and, with increased efficacy, in combination with antibodies blocking PDL-1 [143]. Supported by these preclinical data, a phase I open-label, multicenter clinical study (NCT02764151) on recurrent malignant GBM enrolled seventeen patients. The disease control rate occurred in eight patients (47%) with a mean duration of the stable disease being 32.1 weeks. Four patients experienced serious adverse events, one with treatment-related severe adverse events (AEs) (grade 4 alanine and aspartate aminotransferase elevations) [144]. Other molecules are being studied in monotherapy or in combination with other treatments. Indoximod or 1-methyl-tryptophan was described as an inhibitor of the IDO1 enzyme in the early 1990s [145]. Currently, a phase II study in combination with chemo-radiation is recruiting (NCT04049669). BMS is an oral irreversible inhibitor that reduces kynurenic levels even at low concentrations. A phase I (NCT04047706) study with nivolumab and chemo-radiation in newly diagnosed GBM is recruiting. Epacadostat, a reversible competitive IDO1 inhibitor, is also under study, alone or in combination (NCT03532295).

### 6.2. IDH Mutation Inhibitors

Another of the most investigated strategies is *IDH* inhibitors. Although the *IDH* mutation is mostly found in low-grade gliomas, up to 5% of GBM have it. For this reason, GBM is under-represented in clinical trials with these *IDH* inhibitors, nevertheless, they represent a great opportunity for the treatment of GBM.

Both *IDH1* and *IDH2* inhibitors have been developed in different types of tumors: Ivosidenib, BAY-1436032, and AG-5198 for *IDH1* mutations, and Enasidenib, AGI-6780 and GI-6780 for *IDH2* mutations [146]. Vorasidenib (AG-881) is a pan-inhibitor of both *IDH1/2* mutants. Results from a phase 1 (NCT02481154) safety and dose-escalation trial of Vorasidenib on a non-enhancing cohort were updated at ESMO 2020. 22 gliomas received treatment with AG-881 (dose range: 10–200 mg daily). The most frequently observed AE was the elevation of transaminases, reversible with drug interruption and dose modification. The overall response rates (ORR) were 18.2% (one patient achieved a partial response and three patients achieved a minor response). 72.2% of patients had stable disease as their best response. The median progression-free survival (PFS) was 31.4 months (95% CI, 11.2,40.8) with 59.1% events reported. A Vorasidenib dose of 50 mg QD was selected and is under evaluation in the ongoing INDIGO study, randomized phase 3 in grade 2 non-enhancing *IDH1/2* mutant glioma patients who have undergone surgery only (NCT04164901).

### 6.3. Pyruvate and Lactate Antagonist. Regulation of Hexokinase 2

Pyruvate dehydrogenase (PDH) is an enzyme that metabolizes pyruvate to acetyl CoA and its activity is regulated by reversible phosphorylation. 3-bromopyruvate (3BP) is an organic compound similar in structure to pyruvate and lactate that may antagonize their effects of producing H_2_O_2_ [147]. Furthermore, it is a potent antiglycolytic agent able to inhibit glycolytic and mitochondrial ATP production, and it can inhibit hexokinase 2 [148]. Several studies observed that 3BP interferes with carbohydrate metabolism, depleting ATP levels, causing mitochondrial dysfunction, degrading HK2, and inducing the appearance of autophagic flux markers [147,149,150]. A regulation of HK2 levels has also been described with the use of 5-aza-2-deoxy-cytidine [151]. These findings are important for understanding the mechanisms of 3BP and thus furthering the role of glycolytic inhibitors in the treatment of GB.

### 6.4. Targeting Phospholipids

Polymerase 1 and transcript release factor (PTRF), also known as Clavin-1, a plasma membrane microdomain with several functions in signal transduction [152], has gained relevance in recent years. Increased PTRF expression has been reported to correlate with a worse prognosis in glioma patients and increased tumor cell proliferation and immune suppression in GBM [153]. Yi et al., detected the relationship between PTRF and lipid metabolism in GBM and observed a significant increase in the cytoplasmic phospholipase A2 (cPLA2) protein in GBM cells with PTRF overexpression. In GBM xenografts and intracranial tumor mouse models, they showed that inhibiting cPLA2 activity blocks tumor proliferation and prevents PTRF-induced reduction in CD8 TILs, thus suggesting cPLA2 as an attractive therapeutic target [154].

## 7. Discussion

The GBM TME is complex and consists of different cell types that interact with each other to promote tumor growth. Other factors are involved in this process, such as several metabolic pathways that contribute to maintaining an immunosuppressed environment and regulate progression, treatment response, and disease recurrence. In addition, genetic aberrations, fundamental drivers of GBM tumorigenesis, are associated with metabolic adaptations. A better understanding of the interaction of all these factors is essential for the development of new therapeutic modalities. In this review, we focus on the complexity of the tumor microenvironment and the metabolic diversity in GBM, as well as on therapeutic lines of research.

Targeting metabolic pathways is providing an opportunity to extend the limited, currently approved, treatment options. While there are many ongoing clinical trials exploring immunotherapies with checkpoint blockade in GBM, there are only a few investigating immunometabolism treatments. The tryptophan metabolism pathway is the one of most clinically explored IDO inhibitors with different phase I/II clinical trials, alone or in combination, as well as *IDH1/2* inhibitors in the glutamine pathway. However, many of the other pathways analyzed in this review have not yet been explored or have been poorly explored in the clinic.

The interaction between metabolic mechanisms and TME/immune cells is another potential therapeutic target. The TME of glioma is characterized by a low percentage of TILs and TAMs as the predominant cell providing an immunosuppressive environment, so, to try and create a pro-inflammatory/antitumoral environment that is less permissive for tumor progression is a good strategy.

An immunometabolism research line is difficult because it requires an in-depth understanding of the genetic alterations. The heterogeneity of genetic alterations in GBM represents a major contribution to treatment resistance and promotes an altered metabolism, but it is incompletely understood. Fortunately, over the last decade, technological advances have led to the development of new tools, such as next-generation sequencing to study the genomic landscape, functional models to explore molecular and metabolic pathways or imaging tools to define a metabolic profile. The research focused on targeting metabolic pathways allows the obtainment of more data to design strategies to treat patients with GBM individually.

## 8. Conclusions

The role of the intimate interplay between tumor metabolism, tumor microenvironment, and genetic alterations has received considerable attention over the last several years and is recognized as an important factor involved in growth and progression in glioblastoma. Currently, different strategies are being tested in ongoing clinical trials with limited results. More knowledge is required in order to transfer the benefit to the clinic setting.

## Data Availability

Not applicable.

## Abbreviations

2HG: D2-Hydroxyglutarate; 3BP: 3-bromopyruvate; alpha-KG: Alfa ketoglutarate; AE: Adverse events; AHR: Aryl hydrocarbon receptor; ARG1: Arginase 1; *ATRX*: Alpha thalassemia/mental retardation syndrome X-linked; BBB: Blood-brain barrier; cAMP: Cyclic adenosine monophosphate; *CDKN2A*: Cyclin dependent kinase inhibitor 2A; CERS1-6: Ceramide synthase; CGCA: Chinese Glioma Genome Atlas; CL: classical; CNS: Central nervous system; CpG: cytosine-phosphate-guanine; cPLA2: Cytoplasmic phospholipase A2; EGF: Epidermal growth factor; *EGFR*: Epidermal growth factor receptor; EGFRvIII: EGFR variant III; GBM: Glioblastoma; G-CIMP: island methylator phenotype; GLUT1: Glucose transporter; GS: glutamine synthetase; GSC: Gliomas stem cells; HK2: Hexokinase 2; *IDH*: Isocitrate dehydrogenase; IDO1/2: indoleamine 2,3-dioxygenase ½; iNOS: Nitric oxide synthase; MES: mesenchymal; *MGMT*: methyl-guanine-methyl-transferase; NAD+: nicotinamide adenine dinucleotide; NEU: neural; *NF1*: neurofibromatosis 1; NK: Natural Killer; NLR: neutrophil-lymphocyte ratio; NO: Nitric oxide; *PDGFRA*: platelet derived growth factor receptor alpha; *PIK3CA*: Phosphatidylinositol 4,5-bisphosphate 3-kinase catalytic subunit alpha isoform; PN: Proneural; *PTEN*: Phosphatase and tensin homolog; PTRF: Polymerase 1 and transcript release factor; PVN: Perivascular niche; *RB1*: Retinoblastoma-associated protein 1; S1P: Sphingosine-1-phosphate; SGPP: Sphingosine-1-phosphate phosphatase; S1PR1: Sphingosine-1-phosphate receptor 1; SK: Sphingosine kinase; scRNAseq: Single-cell RNA sequencing; scTHI: single-cell Tumor–Host Interaction; STI1: Stress-inducible protein 1; ROS: Oxygen species; RTK: Receptor tyrosine kinase; TAMs: Tumor-associated macrophages; TCA cycle: tricarboxylic acid cycle; TCGA: Cancer Genome Atlas Research Network; TDO: tryptophan 2,3 dioxygenase; *TERT*: Telomerase reverse transcriptase; TGF-β: Transforming growth factor-β; TILs: Tumor-infiltrating lymphocytes; TLR4: Toll-like receptor 4; TME: Tumor microenvironment; TTFields: Tumor-treating fields; VEGF: Vascular endothelial growth factor.
